# Automated extraction of auditory brainstem response latencies and amplitudes by means of non-linear curve registration

**DOI:** 10.1016/j.cmpb.2020.105595

**Published:** 2020-11

**Authors:** Katrin Krumbholz, Alexander James Hardy, Jessica de Boer

**Affiliations:** a School of Medicine, Hearing Sciences Group, University of Nottingham, United Kingdom; b School of Psychology, University of Nottingham, United Kingdom

**Keywords:** Supra-threshold auditory brainstem responses, Hidden hearing loss, Cochlear synaptopathy, Dynamic time warping, Continuous monotone registration

## Abstract

**Background and objectives:**

Animal results have suggested that auditory brainstem responses (ABRs) to transient sounds presented at supra-threshold levels may be useful for measuring hearing damage that is hidden to current audiometric tests. Evaluating such ABRs requires extracting the latencies and amplitudes of relevant deflections, or “waves”. Currently, this is mostly done by human observers manually picking the waves’ peaks and troughs in each individual response – a process that is both time-consuming and requiring of expert experience. Here, we propose a highly automated procedure for extracting individual ABR wave latencies and amplitudes based on the well-established methodology of non-linear curve registration.

**Methods:**

First, the to-be-analysed individual ABRs are temporally aligned – either with one another or, if available, with a pre-existing template – by locally compressing or stretching their time axes with smooth and invertible time warping functions. Then, the individual latencies and amplitudes of relevant ABR waves are obtained by picking the latencies of the waves’ peaks and troughs on the common (aligned) time axis and combining these with the individual aligned responses and inverse time warping functions.

**Results:**

Using an example ABR data set with a wide range of response latencies and signal-to-noise ratios (SNRs), we test different choices for fitting the time warping functions. We cross-validate the warping results using independent response replicates and compare automatically and manually extracted latencies and amplitudes for ABR waves I and V. Using a Bayesian approach, we show that, for the best registration condition, automatic and manual data were statistically similar.

**Conclusions:**

Non-linear curve registration can be used to temporally align individual ABRs and extract their wave latencies and amplitudes in a way that closely matches results from manual picking.

## Highlights

• We propose a highly automated procedure for extracting latencies and amplitudes of auditory brainstem responses (ABRs) based on curve registration through non-linear time warping.
• We compare different registration conditions using an example ABR data set with a wide range of response latencies and signal-to-noise ratios.
• We demonstrate that the best registration condition closely matched the performance of expert human observers.

## 1 Introduction

In humans, sound-evoked neuronal responses from subcortical, or brainstem, auditory structures can be measured non-invasively with electroencephalographic, or scalp electrodes. Currently, the clinical use of these “auditory brainstem responses” (ABRs) is mostly limited to hearing screening and objective estimation of audiometric thresholds. Animal evidence, however, has suggested that, in future, the clinical role of ABRs may expand to detecting hearing damage not thought to be associated with audiometric loss, such as damage to the cochlear synapses [1], [2], [3], [4], inner hair cells [5,6] or auditory nerve [7], which have been found to manifest in a reduction of auditory-nerve responses to supra-threshold sounds. This has prompted considerable efforts in exploring whether, or to what degree, audiometrically hidden hearing damage also occurs in humans [reviewed in 8,9], and developing suitable tests for its measurement and diagnosis [reviewed in 10]. Most of the tests considered so far have involved ABRs elicited by transient, supra-threshold sounds, such as loud clicks [reviewed in 11].

ABR-based measurement of audiometrically hidden hearing damage, such as synaptopathy, poses a fundamentally different analytical challenge than ABR-based estimation of audiometric thresholds. Whilst ABR-based threshold estimation requires to decide whether a measured response contains a ‘true’ ABR over and above the inherent noise, evaluation of supra-threshold ABRs requires characterization of the responses’ properties, such as wave latencies and amplitudes. Many of the previous studies of human synaptopathy have measured the amplitude of the earliest ABR wave, wave I [e.g., [12], [13], [14]], thought to arise from the auditory nerve, and some have also measured the amplitude of the prominent wave V [e.g., 15–17].

Currently, ABR wave latencies and amplitudes are mostly extracted through manual picking of the waves’ peaks and troughs by human observers. However, manual picking is time-consuming and requires expert experience, which is prohibitive when dealing with large data sets or performing a routine clinical test. This study was aimed at devising a procedure for extracting individual latencies and amplitudes of ABR waves that would require minimal human input. First, we review the strengths and limitations of relevant previous approaches. Based on this, we develop the rationale for the current approach, which involves the well-established methodology of non-linear curve registration [18,19]. Using an example ABR data set from 23 normal healthy subjects comprising 12 different experimental conditions, we examine a range of different registration conditions. For each condition, we compare the automatically picked latencies and amplitudes of waves I and V with corresponding manually picked values. Finally, we use a realistic set of simulated ABR data to explore the effect of signal-to-noise ratio (SNR).

## 2 Background

**Fig. 1:**
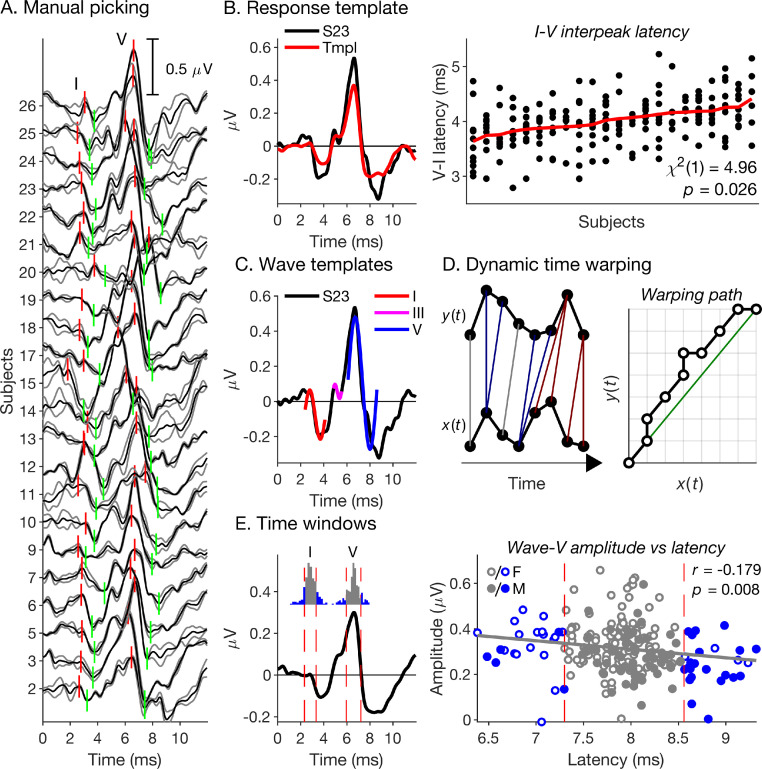
Relevant previous approaches to automated ABR analysis. (A) Example manual picking results (short vertical red and green lines) for waves I and V of a chirp-evoked ABR data set from 22 normal healthy adults (detailed information is given in Section 3.2.1). The black lines show the average evoked responses over all available trials. The grey lines show two response replicates comprising complementary sets of alternate trials. (B) Response template approach proposed by Elberling [22]. Left panel: Example template (red), derived from the responses shown in A, together with a representative individual response (S23; black). Right panel: Manually picked interpeak latencies between waves I and V for a wider data set from the same 22 subjects as shown in A. Each vertical set of dots represents an individual subject's interpeak latencies for up to 12 different experimental conditions. Before plotting, the data were adjusted for any fixed condition effects (by subtracting the subject-average latency difference for each condition, and adding the grand-average latency difference across all conditions and subjects), and ordered by the condition-average latency difference for each subject (red line). A linear mixed model analysis (see Section 3.4), applied to the original latency differences (before adjusting and ordering), showed a significant effect of subjects (see legend). (C) Wave template approach. Left panel: Waves I, III and V of subject S23’s response shown in A were least-squares fitted with truncated sine functions. (D) Dynamic time warping approach proposed by Picton, Hunt, Mowrey, Rodriguez and Maru [33]. Left panel: Schematic illustration of a discrete mapping between two time sequences, *x*(*t*), and *y*(*t*) (black dots and lines). The red and blue lines show instances where the mapping is non-unique. Right panel: Associated time warping path, with *x*(*t*) plotted on the abscissa, and *y*(*t*) plotted on the ordinate. (E) Search window approach used by Prendergast, Guest, Munro, Kluk, Leger, Hall, Heinz and Plack [16]. Left panel: In the current data set, Prendergast et al.’s search windows for the wave-I and -V peaks (dashed vertical lines) contained most, but not all of the respective manually picked peak latencies (see inset histograms). Right panel: The current data showed a significant inverse relationship between the wave-V latency and amplitude (see legend), brought about by differences between males (M) and females (F) [44]. This meant that excluding the shortest and longest wave-V latencies at the edges of the respective search window also excluded the largest and smallest wave-V amplitudes, creating a selection bias towards the group-average amplitude. As for the right panel in B, latencies and amplitudes were corrected for any fixed effects of condition before plotting.

ABRs evoked by transient sounds, such as clicks or brief chirps, consist of a series of consecutive waves [20] arising from different levels of the subcortical auditory pathway [reviewed in 21]. Like other natural time series, ABRs exhibit both amplitude and time variability: features of individual subjects’ ABRs, such as wave peaks or troughs, vary in both amplitude and latency. Due to time variability, the amplitude of a given feature (e.g., wave peak) can only be meaningfully measured after first establishing the feature's latency in each individual response. Otherwise, amplitudes would be measured at different points within the feature's shape and would thus be confounded by variability in its latency. A human observer will pick an ABR wave's vertex-positive peak and the subsequent trough, and take the peak latency as a measure of the wave's latency, and the peak-to-trough amplitude difference as a measure of the wave's amplitude. As example, Fig. 1A shows manual picks of waves I and V in 22 individual ABRs to a broadband chirp stimulus (detailed information about the current data set is given in Section 3.2.1).

To obviate the need for manual picking, Elberling [22] proposed to cross-correlate individual ABRs with a “response template” (Fig. 1B, left panel) to obtain individual measures of overall response latency and response-to-template similarity. The template was generated by averaging the individual responses after time-shifting them to align their wave-V peaks (typically the largest peaks in the responses; see Fig. 1A) and normalizing them by their root-mean-square amplitudes. The template may be considered an estimate of the individual responses’ common underlying structure – a kind of “structural average” [23] response, which, unlike the conventional “cross-sectional” average (i.e., average of the unaligned responses), is less affected by the smoothing effects of time variability. However, as the approach only aligns the wave-V peaks, smoothing could continue to affect other waves, if their interpeak latencies relative to wave V vary across responses. Using the current data set as example, the right panel in Fig. 1B shows that, even in normal healthy subjects of similar age, the interpeak latency between waves I and V can vary significantly across subjects. Even greater variability is known to arise as a result of maturation and ageing [24] and in certain neurological conditions [25].

Two different approaches have been proposed to address this problem. The computationally simpler, but also less powerful approach is the “wave template” approach [[26], [27], [28], [29]], which fits separate templates to individual ABR waves (see Fig. 1C, for a simplified example). Unlike the response templates of Elberling [22], the wave templates are not derived from the measured responses, but implemented as synthetic functions, representing the waves’ idealized shapes. To fit them, each template is allowed to shift and scale in time and in amplitude. This assumes that ABR waves are “shape-invariant”, that is, that they retain the same basic shape across individuals and/or conditions, varying only in size and/or scale. This assumption, however, is unlikely to be generally valid, given that ABR waves represent a mixture of multiple activities generated by different sources [20,[30], [31], [32]], which may vary both in strength and in timing, and thus represents a limitation of the wave template approach.

In contrast, the dynamic time warping (DTW) approach proposed by Picton, Hunt, Mowrey, Rodriguez and Maru [33] makes no assumptions about response shapes – like the response template approach of Elberling [22], it is completely non-parametric. The main difference is that, rather than using linear time shifts to align individual responses, the DTW approach uses non-linear time transformations, locally stretching or compressing portions of the responses to align common features, such as waves (Fig. 1D). Even though the DTW approach was not specifically designed for extracting wave latencies and amplitudes, its basic idea of non-linearly aligning individual responses to form a structural average would seem to hold great promise for achieving this purpose. However, in its original implementation, the approach used the discrete form of time warping, developed previously by Sakoe and Chiba [34] for measuring similarity between speech tokens, rather than the continuous form developed only later [18,19]. As a result, the approach suffered from unnecessary conceptual and computational complexity.

Neither the template-based approaches nor the DTW approach have so far been applied in ABR studies of human synaptopathy. Instead, several of these studies have used a much simpler approach, picking ABR peaks and troughs by finding the corresponding zero-crossings in the temporal derivative of the response waveform [16,17,35,36]. Various derivative-based ABR analysis procedures have been proposed previously [[37], [38], [39], [40], [41], [42], [43]], and all were particularly focussed on separating the ‘true’ ABR from the inherent noise, as noise is disproportionately amplified by differentiation. In contrast, the human synaptopathy studies have dealt with the noise problem by limiting the search for derivative zero-crossings to predefined time windows around relevant wave peaks and troughs in the average response across individuals (Fig. 1E, left panel). Individual responses that did not contain a derivative zero-crossing within a given search window were assumed not to exhibit the corresponding wave and thus excluded from further analysis. This, however, is not necessarily true. Rather, at least a proportion of the excluded responses will have exhibited the relevant wave, but its latency will have lain outside of the respective search window (see insets in Fig. 1E, left panel), and so, exclusion will have created a selection bias towards the wave's group-average latency. If there was an association between the wave's latency and amplitude, the bias would have also affected the amplitudes, and thus potentially created a false null result. In the current data set, the wave-V latency and amplitude exhibited such association (Fig. 1E, right panel) – in this case, created by the effect of gender [44,45] rather than synaptopathy. As a result, imposing the same search windows as Prendergast, Guest, Munro, Kluk, Leger, Hall, Heinz and Plack [16] would have excluded significantly more females than males at the lower end of the latency range (18 vs 2; binomial test: *p* < 0.001), and significantly more males than females at the higher end of the range (22 vs 4; *p* < 0.001), and this would have reduced the wave-V amplitude difference between males and females by a substantial 21%.

## 3 Materials and methods

### 3.1 Design considerations

The previous discussion highlights the need for a new, more specific procedure for extracting ABR wave latencies and amplitudes. Like the response template [22] and DTW [33] approaches, the new procedure should align individual ABRs to create a time-variability-adjusted “structural average” [23] response and define a common “structural” time axis, comparable to the standardized spatial coordinate systems used for aligning medical imaging data [46]. Relevant waves could then be identified in the structural average response, and their peak and trough latencies determined on the common structural time axis (referred to as “structural latencies”). The individual wave amplitudes could then be determined by evaluating the aligned individual responses at the structural peak and through latencies (to yield individual peak and trough amplitudes, although alternative amplitude measures, such as the peak area, could also be derived), and the individual wave latencies could be determined by transforming the structural peak latencies back to the original (unaligned) individual time axes.

As mentioned in Section 2, the DTW approach [33] used a discrete time warping procedure. Such procedures are designed to align sequences of elements that are not amenable to averaging or interpolation, particularly, non-numerical elements, such as amino acids in proteins or nucleotides in DNA and RNA [47]. As a result, discrete time warping is an inherently pairwise procedure, and generalizing it to find the structural average of a set of multiple sequences, as would be required here, is not a trivial task [48]. Moreover, discrete time warping does not allow time-scaling of segments within the to-be-aligned sequences. Instead, single elements in one sequence are allowed to map to multiple elements in the other (Fig. 1D, left panel), and thus the warping path (Fig. 1D, right panel), defining the transformation from the original to the aligned sequences, is non-invertible. When applied to ABRs, this would preclude transforming the structural latencies (on the aligned time axis) back to the original (unaligned) time axes.

**Fig. 2:**
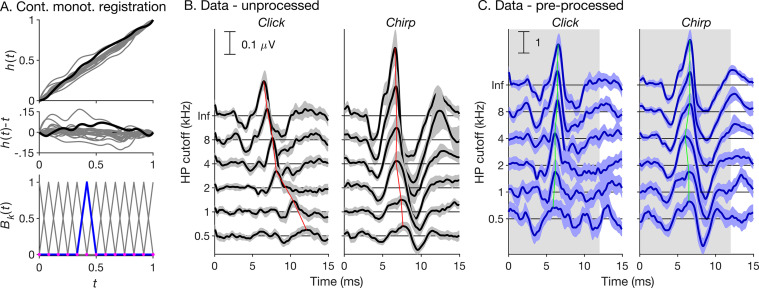
Continuous monotone registration approach [18] and test data. (A) Top and middle panels: Example time warping functions, *h*(*t*), constructed using the continuous monotone registration approach, and corresponding differences, *h*(*t*)−*t*, with the original time axis, *t*. Bottom panel: Linear B-spline functions (grey lines) used to model the logarithm of the first derivative of the warping functions, ln{*h*′(*t*)}. The thick blue line highlights one example B-spline. The purple dots represent the knots (see Section 3.3.1). (B) ABR data set used to test the proposed procedure. The left and right panels show different stimulus conditions (click and chirp). The horizontally staggered lines show different high-pass masking conditions (the ordinate shows the high-pass masker cutoff frequency in kHz; “Inf” signifies the broadband condition). The black lines show the average responses across subjects for each condition, and the grey-shaded areas show the corresponding cross-subject 95% confidence ranges. The thin red line connects the wave-V peak latencies across conditions. (C) Same data as in B, but after pre-processing (pre-alignment across high-pass masking conditions, cross-fading between 100- and 150-Hz high-pass-filtered versions, and normalization for root-mean-square amplitude; see Section 3.2.2). The grey highlight shows the warping time range (0–12 ms).

Therefore, we here propose to use a continuous implementation of dynamic time warping [18,19], also known as “(non-linear) curve registration”. Curve registration is part of functional data analysis [49] and deals with sequences of continuous numerical data that, like ABRs, can be regarded as discretizations of smooth functions, or “curves” (rather than sequences of discrete elements, such as proteins). In the continuous case, the warping functions (equivalent to the warping paths in the discrete case) are specifically designed to be not only smooth, but also strictly increasing (see Fig. 2A, upper and middle panels). This ensures that single points in the original curves are uniquely mapped to single points in the structural average curve and vice versa (i.e., the warping functions are invertible). To construct such functions, we here opted for the “continuous monotone registration” method proposed by Ramsay [50], which models the logarithm of the warping functions’ derivative as a linear combination of B-spline, or “basis-spline” functions (Fig. 2A, lower panel; see Section 3.3.1).

To test the approach, we applied it to extract the wave-I and -V latencies and amplitudes from an example ABR data set comprising 23 normal healthy subjects and 12 different experimental conditions (Fig. 2B). To create a gold-standard reference, the same latencies and amplitudes were also extracted manually by three expert observers (the three authors), who were blinded to subjects and conditions.

### 3.2 Data acquisition and pre-processing

#### 3.2.1 ABR acquisition

ABRs were recorded from a total of 23 (14 female, 9 male) young (age range = 20–37 years), normal-hearing (thresholds in the test ear ≤ 25 dB HL at all octave frequencies between 0.125 and 8 kHz) subjects, none of whom reported any history of audiological or neurological disease, and who gave prior written informed consent. The study procedures complied with the Declaration of Helsinki guidelines (version 6, 2008) and were approved by the Ethics Committee of the University of Nottingham School of Medicine, but were not formally pre-registered as stipulated by the Declaration's 2014 amendment.

The evoking stimulus was either a click, or a chirp designed to compensate for cochlear dispersion. The click and chirp had the same energy level, corresponding to a peak-equivalent sound pressure level (SPL) of 90 dB for the click. They were generated by adding sinusoids at integer multiples of 10 Hz between 0.25 and 8 kHz (with rounded spectral edges to avoid edge tones) either in cosine phase (for the click) or with the phase delays proposed by Elberling and Don [51] (for the chirp). They were presented either in quiet (referred to as “broadband” condition), or in a background of continuous high-pass-filtered noise (referred to as “high-pass-masked” conditions). The noise was filtered from an 80-dB SPL white noise using one of five different octave-spaced cutoff frequencies (0.5, 1, 2, 4 or 8 kHz). The noise is intended to mask any response contributions above the cutoff frequency. Normally, high-pass masking is used to estimate response contributions from narrow frequency regions [so-called “derived-band” responses; 52]. Here, masking was merely used to generate responses with a wide range of latencies and SNRs. The stimuli were presented with alternating polarity at a rate of 20/s and repeated for a total of at least 6000 times for each condition.

The stimuli and the noise were generated digitally (24.4-kHz sampling rate) in Matlab (version R2019a, The Mathworks, Natick, MA, USA), digital-to-analogue converted (24-bit amplitude resolution) using Tucker Davis Technologies (Alachua, FL, USA) System 3 (RP2.1 and HB7) and presented monaurally to the left ear via ER-2 insert earphones (Etymotic Research Inc., Elk Grove Village, IL, USA).

The ABRs were acquired using a BioSemi ActiveTwo system (BioSemi B. V., Amsterdam, Netherlands) with ABR-type electrodes and a vertical montage, with reference at the vertex, active electrode on the ipsilateral mastoid, and ground on the forehead. They were sampled at a rate of 16.384 kHz.

#### 3.2.2 ABR pre-processing

All pre-processing was performed using Matlab R2019a. First, the raw data were low-pass filtered at 2 kHz and high-pass filtered at 100 and 150 Hz (both the low- and high-pass filters were implemented as 4th-order Butterworth IIR filters), and divided into 45-ms epochs including a 5-ms pre-stimulus baseline. Then, the 6000 least artefactual epochs (in terms of overall maximum voltage) were submitted to a Bayesian weighted-averaging procedure [using 250-trial blocks; 53] to create an overall average response for each condition and subject, as well as two replicate responses comprising alternate halves (3000) of the trials. The Bayesian-averaged responses were then further averaged across subjects to create a “cross-sectional” average response for each condition.

Fig. 2B shows that, particularly for the click stimulus, the average responses for different high-pass-masking conditions differed substantially in overall latency. This is due to cochlear dispersion [54]. Such average condition-related latency differences should be eliminated by linear time shifting before trying to non-linearly align the individual responses [18]. Here, we advanced the high-pass-masked responses to coincide with the earlier broadband response (Fig. 2C). The shift delays were based on the average responses across subjects. They were determined by cross-correlating the responses and finding the lag that maximized their correlation coefficient for the time range between 0 and 12 ms.

Next, we combined the 100- and 150-Hz high-pass-filtered versions of the responses by cross-fading them with 2-ms linear transition ramps centred on 5 ms. As a result, the combined responses contained the 150-Hz-filtered version in the time range of wave I (≤ 4 ms), and the 100-Hz-filtered version in the time range of wave V (≥ 6 ms), as we found these filter settings to be optimal in pilot tests [see also 55]. Finally, each individual response was mean-corrected and normalized by its root-mean-square amplitude over the time range from 0 to 12 ms (compare scale bars in Figs 2B and 2C).

### 3.3 Automatic extraction of ABR wave latencies and amplitudes

#### 3.3.1 Construction of time warping functions

Time warping was performed within the time range between 0 and 12 ms (grey highlight in Fig. 2C), which contained both the peaks and subsequent troughs of waves I and V in all pre-processed responses. For convenience, this was normalized to range from 0 to 1 (see Fig. 2A). To align a given individual response, *x_i_*(*t*) (where *i* indicates the individual and *t* indicates time), with a given target, *y*(*t*), a time warping function, *h_i_*(*t*), was constructed by modelling the natural logarithm of its first derivative, ln {*h_i_*′(*t*)}, as a linear combination, $w_{i}\left(t\right)=\sum_{k=1}^{K}c_{ik}B_{k}\left(t\right)$, of 2nd-order (linear) B-spline functions, *B_k_*(*t*), with *K*equally spaced knots, *t_k_* = (*k* − 1)/(*K* − 1) = (*k* − 1)Δ*t*, where Δ*t* = 1/(*K* − 1) is the knot spacing (see Fig. 2A, lower panel). *K* was set to 13, so that Δ*t* corresponded to 1 ms. The B-splines were defined as:

$$B_{k}\left(t\right)=\left\{ \begin{array}{c} \left(t-t_{k-1}\right)/\Delta t,\;t\in \left[t_{k-1}\;t_{k}\right]\\ \left(t_{k+1}-t\right)/\Delta t,\;t\in \left[t_{k}\;t_{k+1}\right]\\ 0\;otherwise \end{array}\right.$$

Note that, for *k* = 1, *B_k_* is not defined over [*t*_*k* − 1_ *t_k_*], and for *k* = *K, B_k_* is not defined over [*t_k_* *t*_*k* + 1_]. In order to derive *h_i_*(*t*), *w_i_*(*t*) was submitted to the exponential function, integrated, and then scaled and shifted, so that *h_i_*(*t*) spanned the same range as *t* ([0 1]): $h_{i}\left(t\right)=A_{i}+B_{i}\int_{0}^{t}\text{exp}\left\{w_{i}\left(u\right)\right\}du$. The B-spline coefficients, ***c**_i_* = [*c*_*i*1_, *c*_*i*2_, …,  *c_iK_*], were fitted by minimizing a suitable cost function (Section 3.3.2) using the constrained non-linear problem solver *fmincon*in Matlab with the default interior-point algorithm.

Time warping was performed on the discrete ABR time series using Matlab R2019a. Derivatives were approximated by Newton's difference quotient, and integrals by the rectangle rule. The warped responses, $x_{i}^{*}\left(t\right)=x_{i}\left\{h_{i}\left(t\right)\right\}$, were created by evaluating the original responses, *x_i_*(*t*), at the warped time points, *h_i_*(*t*), using linear interpolation.

#### 3.3.2 Choice of fitting criterion

In its original version, the continuous monotone registration method fits the B-spline coefficients by minimizing a penalized squared difference criterion, $PSD\left(c_{i}\right)=\int_{0}^{1}|y\left(t\right)-x_{i}^{*}\left(t\right){|}^{2}dt+\lambda \int_{0}^{1}{w_{i}}^{\prime }{\left(t\right)}^{2}dt$. Here, the first term is the sum of squared differences between the warped and target curves, $x_{i}^{*}\left(t\right)$and *y*(*t*), and the second term is a warping roughness penalty, which shrinks the derivative, *w_i_*′(*t*), of the fitted spline function, *w_i_*(*t*) (see Section 3.3.1), according to a smoothing parameter, λ. Here, we tested five different values of λ, including λ = 0 (no roughness penalty) and four non-zero values, ranging from 0.001 to 1 in decade steps. As the spline function models the logarithm of the derivative of the warping function, ln {*h_i_*′(*t*)}, its derivative represents the warping function's relative curvature, *h_i_*′′(*t*)/*h_i_*′(*t*), [50].

**Fig. 3:**
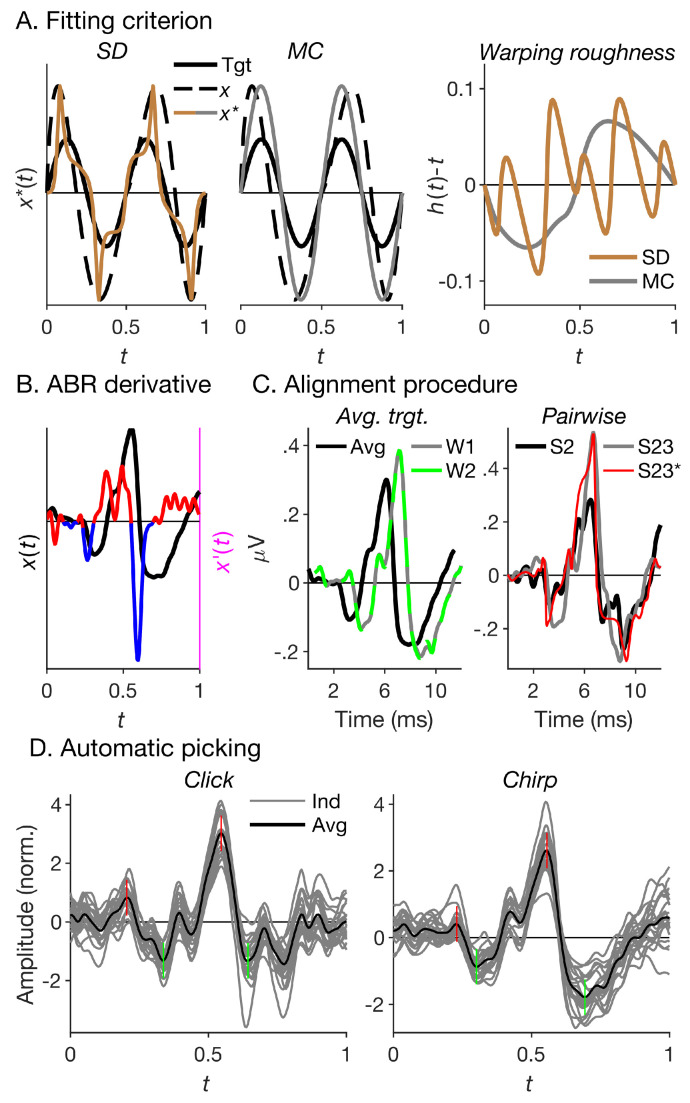
Fitting criteria, registration procedure and automatic picking. (A) Left panel: The squared difference (SD) fitting criterion can lead to the warped curve (x*; brown line) being distorted when the to-be-warped (*x*; black dashed line) and target (Tgt; black solid line) curves differ in amplitude. Middle panel: The maximum correlation (MC) criterion avoids distortion in x* (grey line) when the amplitude difference is constant over time. Right panel: The warping roughness penalty controls the relative curvature of the warping function, *h*(*t*), illustrated here as *h*(*t*)−*t*. Curvature tends to be greater for distorting (brown) than non-distorting (grey) warping functions. (B) Ramsay and Li [18] proposed to register the curve derivatives, *x*′(*t*) (red/blue line; right ordinate), rather than the original curves, *x*(*t*) (black line; left ordinate). The derivative indicates where a curve rises (red) and falls (blue). (C) Left panel: In the average-target (*at*) registration procedure, the average of the first-warped responses (W1; grey solid line) differed substantially from the average of the original responses (“Avg”; black solid line), but little from the average of the second-warped responses (W2; green dashed line). Here, this is illustrated using the broadband chirp-evoked ABRs shown in Fig. 1A. Right panel: Illustration of the pairwise (*pw*) registration procedure [58], which warps each individual response (current example: S23; grey and red lines) to every other individual response (current example: S2; black line). (D) Individual wave-I and -V latencies and amplitudes were extracted by finding the latencies of the waves’ peaks and troughs (short vertical red and green lines) in the structural grand-average response (average aligned response across subjects and high-pass-masking conditions) for each stimulus condition (click/chirp; bold black lines). The thin grey lines show the corresponding individual responses.

The squared deviation shrinks the pointwise amplitude differences between the warped and target curves. If the curves, or some of their features, differ in amplitude, this can lead to shape distortion by shrinking time periods where amplitude differences are large, and expanding periods where amplitude differences are small. An example of shape distortion is shown in the left panel of Fig. 3A. The roughness penalty can ameliorate this problem, as shape distortion tends to be associated with high warping function curvature, or roughness (Fig. 3A, right panel), but rarely eliminates the problem entirely. Other means of reducing shape distortion include reducing amplitude differences between the to-be-aligned and target curves [19,56] – here, we normalized the individual ABRs by their root-mean-square amplitude (see Section 3.2.2) – and using a different fitting criterion [18]. Here, we tested a total of four different criteria, including the original *PSD* criterion, as well as three alternative criteria. The first alternative criterion, the penalized squared difference of the derivatives, or *PSDD*, criterion is similar to the *PSD* criterion, except that it minimizes the sum of squared differences between the curves’ derivatives (Fig. 3B), $\int_{0}^{1}|y^{\prime }\left(t\right)-x{{}_{i}^{*}}^{\prime }\left(t\right){|}^{2}dt$, rather than the curves themselves. The second alternative criterion, the penalized maximum correlation, $PMC=-\rho \left(y\left(t\right),x_{i}^{*}\left(t\right)\right)+\lambda \int_{0}^{1}{w_{i}}^{\prime }{\left(t\right)}^{2}dt$, maximizes the Pearson correlation, *ρ*, between the warped and target curves, rather than minimizing their squared differences. As the Pearson correlation is independent of overall amplitude differences, correlation maximization may be more appropriate for problems like time warping, where a ‘perfect’ fit does not necessarily mean zero deviation between the warped and target curves [Fig. 3A middle panel; 57]. Finally, for completeness, we also tested the penalized maximum correlation of the derivatives, or *PMCD*, criterion, which is similar to the *PMC*criterion except that it maximizes the correlation of the curves’ derivatives, $\rho (y^{\prime }\left(t\right),x{{}_{i}^{*}}^{\prime }\left(t\right))$. All four fitting criteria (*PSD, PSDD, PMC*, and *PMCD*) were paired with all five values of the smoothing parameter, λ (0, 0.001, 0.01, 0.1 and 1), yielding a total of 20 loss functions.

#### 3.3.3 Choice of registration procedure

To align a set of individual curves, *x_i_*(*t*), *i* = 1, 2, …, N, and construct their structural average (average of aligned curves), $x^{*}\left(t\right)=\frac{1}{N}\sum_{i=1}^{N}x_{i}^{*}\left(t\right)$, the continuous monotone registration method first warps the original curves (*x_i_*(*t*)) to the cross-sectional average curve, $x\left(t\right)=\frac{1}{N}\sum_{i=1}^{N}x_{i}\left(t\right)$, and then iterates the process, using the structural average curve (x*(*t*)) as new warping target. The process is typically stopped after the first iteration, as the average of the first-warped curves usually differs little from that of the second-warped curves. This was also true for the current data (Fig. 3C, left panel). This is the so-called “average target” (*at*) registration procedure.

In addition, we also tested the “pairwise” (*pw*) registration procedure proposed by Tang and Müller [58], which aligns each curve, *x_i_*(*t*), with every other curve, *x*_*j* ≠ *i*_(*t*) (see Fig. 3C, right panel, for an example), and then takes the average of the resulting warping functions $h_{i}^{\left(j\neq i\right)}\left(t\right)$as the curve's overall warping function, $h_{i}\left(t\right)=\frac{1}{N-1}\sum_{j\neq i}h_{i}^{\left(j\right)}\left(t\right)$. Tang and Müller suggested that *pw*registration may be less prone to shape distortion than *at* registration. Both procedures were paired with all of the 20 loss functions to yield a total of 40 registration conditions.

#### 3.3.4 Registration protocol and extraction of wave latencies and amplitudes

Registration was performed separately for each stimulus condition (click or chirp), but collectively for the different high-pass-masking conditions, using the broadband condition (with the highest SNR) as common warping target. For the *at* registration procedure, each individual ABR for each condition was first warped to the average broadband response, and then the resulting warped responses were warped again to average of the first-warped broadband responses. For the *pw* registration procedure, each individual response for each condition was warped to each of the other individuals’ responses for the broadband condition and the resulting warping functions averaged.

All registrations were repeated three times – once for the overall individual responses (based on all 6000 trials; see Section 3.2.2), and again for each of the two replicate responses (based on alternate sets of 3000 trials). The warping functions resulting from the replicate registrations, $h_{i}^{\left(1\right)}\left(t\right)$and $h_{i}^{\left(2\right)}\left(t\right)$, were used to create “same-warped” and “cross-warped” replicates by applying them either to the replicates used for fitting them (e.g., $x_{i}^{*(11)}\left(t\right)=x_{i}^{\left(1\right)}\left\{h_{i}^{\left(1\right)}\left(t\right)\right\}$), or to the respective other replicates (e.g., $x_{i}^{*(21)}\left(t\right)=x_{i}^{\left(2\right)}\left\{h_{i}^{\left(1\right)}\left(t\right)\right\}$). The registrations of the overall responses were used to extract the latencies and amplitudes of waves I and V – the waves used in the human synaptopathy literature (see Section 1). For that, we picked the latencies of the waves’ peaks and troughs, *t_peak_*and *t_trough_*, in the structural “grand-average” response for each stimulus condition (that is, the average of the structural average responses across all high-pass-masking conditions, Fig. 3D; alternatively, we could also have used the structural average responses for the broadband condition only). To estimate the waves’ amplitudes, we took the amplitudes of the aligned individual responses at these latencies, $x_{i}^{*}\left(t_{peak/trough}\right)$, and calculated their peak-to-trough differences. And to obtain the waves’ latencies, we evaluated the individual warping functions at the structural peak latencies (*h_i_*(*t_peak_*)) using linear interpolation, and then transformed back to milliseconds (as *t*and *h_i_*(*t*) were normalized between 0 and 1) and added the correlation lags between the average high-pass-masked and broadband responses, which had been used for linearly pre-aligning the responses at the pre-processing stage (see Section 3.2.2).

### 3.4 Statistics

A linear mixed model analysis of the manually picked latency differences between waves I and V was performed using the *nlme* package [59] for *R* [60]. The model included a fixed condition factor (12 levels) and a random subject intercept. The significance of the subject effect was tested using the likelihood ratio method.

A Bayes factor analysis comparing the automatically and manually picked wave-I and -V latencies and amplitudes was performed using the *BayesFactor* package for *R* [61]. The analysis was applied separately to each stimulus condition (click/chirp) and each data type (latencies/amplitudes). For each condition and data type, the Bayes factor was computed between the most likely model that included only wave (I or V) and high-pass-masking condition (0.5, 1, 2, 4, 8 and inf kHz) as fixed factors and the most likely model that additionally included the factor of picking method (manual vs automatic). Subjects were included as random intercepts.

## 4 Results

### 4.1 Alignment strength

**Fig. 4:**
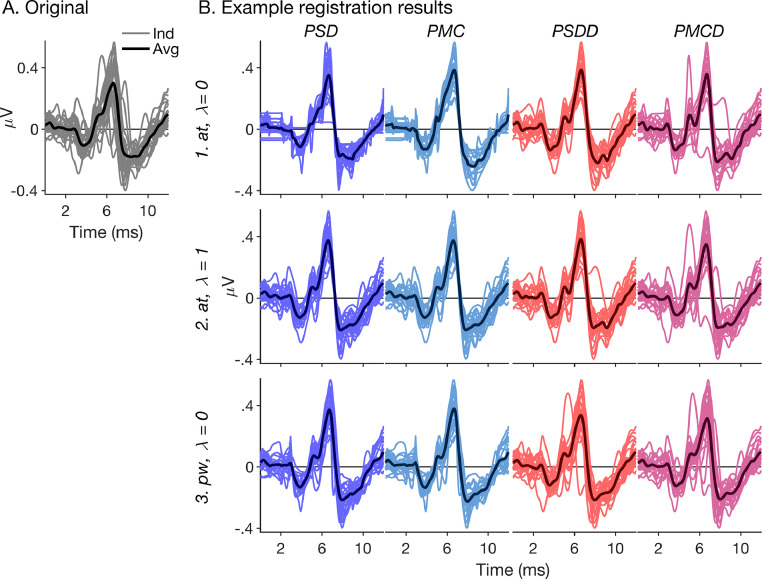
Example registration results for the broadband chirp-evoked ABRs. (A) Original (unaligned) responses (the same as shown in Fig. 1A). (B) Aligned responses. The columns show the four tested fitting criteria (*PSD, PMC, PSDD*, and *PMCD*; see Section 3.3.2) and the rows show three example combinations of registration procedure [average-target (*at*), or pairwise (*pw*)] and smoothing parameter, *λ*. The thinner, lighter-shaded lines show the individual responses, and the thicker, darker-shaded lines show their averages.

Fig. 4 shows example registration results for a representative subset of registration conditions, illustrated using the individual ABRs for the broadband chirp condition. Panel A shows the original (unaligned) responses, replotted from Fig. 1A. Panel B suggests that most registration conditions created at least some degree of response alignment, in that the aligned individual responses were generally more tightly clustered around their average than the original responses. However, the figure also suggests that the strength of the alignment effect varied across conditions, and that some conditions caused shape distortion. Specifically, for the *PSD* and *PMC* fitting criteria combined with the average-target (*at*) registration procedure and no roughness penalty (smoothing parameter, *λ* = 0), it is apparent that some response portions were unduly stretched, resulting in unnaturally flat plateaus, whilst some portions were unduly compressed, resulting in unnaturally sharp peaks or troughs.

**Fig. 5:**
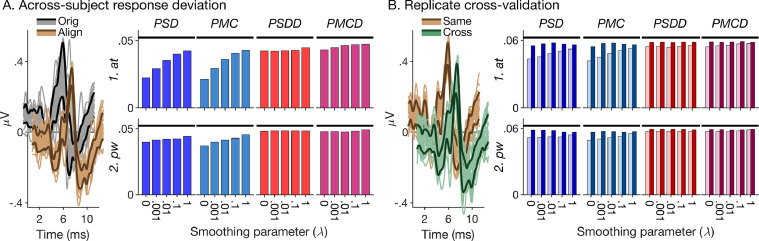
Alignment strength and replicate cross-validation. (A) Response deviation across subjects. Left panel: Illustration of method. The thin lighter-coloured lines show the individual original (grey) and aligned (brown) responses for the broadband chirp condition (same as shown in Figs. 1A and 4). The thicker darker-coloured lines show the responses’ pointwise standard deviation across subjects added to, and subtracted from the response average. The responses were offset along the abscissa and ordinate for clarity. In this example, the registration was performed using the average-target (*at*) procedure and the *PSDD* fitting criterion with no roughness penalty (*λ* = 0). Right panel: Response deviations for all tested registration conditions (coloured bars). The black horizontal lines show the response deviation of the original responses for comparison. (B) Cross-validation based on the response replicates. Left panel: Illustration of method. Pointwise standard deviation across subjects of the same- (brown) and cross-warped (green) replicate responses, plotted in the same way as in A. Right panel: Same- and cross-warped response deviations (lighter- and darker-shaded bars, respectively) for all tested warping conditions. The black horizontal lines show the response deviation of the original replicate responses.

To quantify alignment strength, we calculated the original and aligned responses’ pointwise standard deviations across subjects (Fig. 5A, left panel), after mean-correcting and root-mean-square amplitude normalizing the responses over the warping time range (0–12 ms). To create a single-valued measure for each warping condition, henceforth referred to as “response deviation” (Fig. 5A, right panel), we then averaged the pointwise deviations over time (0–12 ms) and across all experimental conditions. The response deviations of the aligned responses were consistently smaller than those of the original responses, confirming that all tested registration conditions created at least some degree of alignment. Generally, the fitting criteria based on the ABR waveforms (*PSD* and *PMC*) created stronger alignment (smaller response deviations) than those based on the ABR derivatives (*PSDD* and *PMCD*), and the fitting criteria involving squared-difference minimization (*PSD* and *PSDD*) created stronger alignment than those involving correlation maximization (*PMC* and *PMCD*). Response deviations were also generally smaller (indicating stronger alignment) for the average-target (*at*) than pairwise (*pw*) registration procedure. Finally, response deviations tended to increase with increasing smoothing parameter, *λ*, but only for those conditions that yielded small response deviations (strong alignment) when *λ* was zero, namely the *PSD* and *PMC* fitting criteria, particularly when combined with the *at* registration procedure. In contrast, the response deviations for the *PSDD* and *PMCD* fitting criteria depended little on *λ*.

### 4.2 Replicate cross-validation

The response deviations shown in Fig. 5A are based on the overall responses for each subject and condition (based on all 6000 trials; see Section 3.2.2). They measure overall alignment strength, but do not indicate whether the responses were over-aligned: when warping functions are fitted and applied to the same responses, it is possible that not only the ‘true’ ABRs are aligned, but also the inherent noise. To examine whether, or to what degree, this applied to the tested registration conditions, we also calculated the response deviations of the same- and cross-warped replicate responses (Fig. 5B, left panel; Section 3.3.4). Replicates contain the same ‘true’ ABR but independent noises [62]. Consequently, an over-aligning warping function should align both the ABR and the noise in the same-warped replicates, but only the ABR in the cross-warped replicates. Thus, the difference between cross- and same-warped response deviations should indicate the degree of noise alignment. Conversely, the difference between original and cross-warped response deviations should indicate the degree of alignment of the ‘true’ ABR.

Fig. 5B (right panel) suggests that those registration conditions that created the strongest alignment of the overall responses also created strong noise alignment of the replicates (large differences between the cross- and same-warped response deviations). This was particularly apparent for the *PSD* and *PMC* fitting criteria combined with the *at* registration procedure and no or little roughness penalty (*λ* near zero). In contrast, the conditions that created the weakest alignment of the overall responses created little noise alignment (small differences between cross- and same-warped response deviations), but also little ABR alignment (similarly small differences between original and cross-warped response deviations) of the replicates. This was true for the *PSDD* and *PMCD* fitting criteria combined with the *pw* registration procedure, irrespective of *λ*. Some conditions, however, which created intermediate alignment of the overall responses, seemed to create at least some degree of ABR alignment of the replicate responses, whilst, at the same time, not causing too much noise alignment. This applied especially to the *PSD* and *PMC* fitting criteria combined with the *at* registration procedure and a strong roughness penalty (*λ* close to unity) and, to a slightly lesser degree, the *PSDD* and *PMCD* fitting criteria combined with the *at* registration procedure, irrespective of *λ*.

### 4.3 Comparison between automatic and manual picking results

**Fig. 6:**
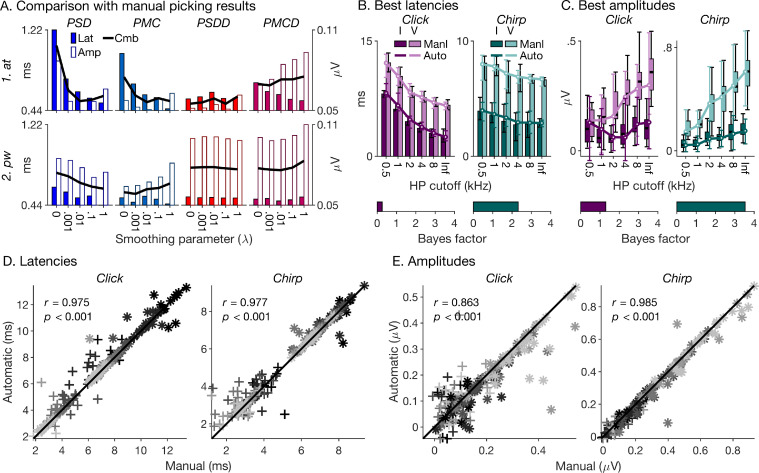
Comparison between automatically and manually picked wave-I and -V latencies and amplitudes. (A) Root-mean-square deviations between automatically and manually picked latencies (filled bars, left ordinate) and amplitudes (open bars, right ordinate) across waves, experimental conditions and subjects. The thick black lines show the combined deviations, which were minimal for the *at, PSDD, λ* = 0 condition. (B) Median manually and automatically extracted latencies for each wave (I and V) and experimental conditions. The automatic latencies are for the *at, PSDD, λ* = 0 condition. The manual latencies are shown as bars, and the automatic latencies as lines. The darker shades show the wave-I results, and the lighter shades the wave-V results. The error bars show the range between the first and third quartiles (*q25, q75*) minus and plus 1.5 times the interquartile range (*iqr*), respectively (*q25*–1.5⋅*iqr* and *q75*+1.5⋅*iqr*). (C) Median manually and automatically extracted amplitudes for the same registration condition as the latencies in B (*at, PSDD, λ* = 0). The manual amplitudes are shown as boxes, and the automatic amplitudes as lines. The height of the boxes shows the range between *q25* and *q75*, and the error bars show the range between *q25*–1.5⋅*iqr* and *q75*+1.5⋅*iqr*. Each bottom inset in B and C shows the Bayes Factor between the most likely statistical model including only the effects of wave and high-pass-masking condition, and the most likely model also including the effect of picking method. Effectively, the Bayes Factors show the likelihood of the null hypothesis that the manually and automatically picked latencies and amplitudes were statistically similar. (D) Scatterplot of manual and automatic latencies for the click (left panel) and chirp (right panel) stimulus. Different waves are shown by different symbols (*I* = +; *V* = *) and different high-pass masking conditions by different shades (lighter shades indicate higher cutoff frequencies). The legend gives the Pearson correlation coefficient. (E) Same as D, but for the amplitudes.

Here, we examine how well the tested registration conditions were able to replicate our manual picking results for the wave-I and -V latencies and amplitudes. To quantify the correspondence between automatically and manually picked data, we calculated their root-mean-square deviations across waves and experimental conditions (Fig. 6A). Both the latency and amplitude deviations showed considerable variation across registration conditions, yet, large latency deviations were not necessarily associated with large amplitude deviations and vice versa. Large latency deviations were particularly associated with registration conditions that created over-alignment (strong noise alignment), namely, the *PSD* and *PMC* fitting criteria combined with the *at* registration procedure and no or little roughness penalty (*λ* near zero). This is understandable, given that over-alignment is associated with over-warping of the individual time axes, and thus misestimation of individual latencies. In contrast, large amplitude deviations were particularly associated with registration conditions that caused under-alignment (little noise, but also little ABR alignment), namely, the *PSDD* and *PMCD* fitting criteria combined with the *pw* registration procedure, irrespective of *λ*. Again, this is understandable: in the extreme case where a registration condition fails to cause any alignment at all, individual amplitudes would be measured at a constant latency across subjects, and would thus deviate substantially from their actual values. The smallest combined latency and amplitude deviations were yielded by the registration conditions that created intermediate alignment effects (reasonable ABR alignment and not too much noise alignment), particularly the *PSDD* fitting criterion combined with the *at* registration procedure, across all values of *λ*. The overall smallest combined deviation was associated with the *PSDD, at* and *λ* = 0 condition.

Fig. 6B shows that, despite the large differences in amplitude, and thus SNR, between waves and experimental conditions, the match between automatically and manually picked latencies and amplitudes was remarkably good across both conditions and waves. The correspondence was formally tested with a Bayes Factor analysis comparing the most likely statistical models excluding and including the effect of picking method (manual vs automatic) for each stimulus (click/chirp) and data type (latencies/amplitudes; see Section 3.4). All Bayes factors were either greater than, or close to, unity, and thus did not provide compelling evidence against the null hypothesis that the manually and automatically picked latencies and amplitudes were not statistically different. This is consistent with the finding, depicted in Fig. 6D and E, that manually and automatically picked latencies and amplitudes were highly significantly correlated. At the same time, Bayes factors were generally larger (indicating a better match between automatically and manually picked data) for the chirp than for the click stimulus. Presumably, this is, because the chirp yielded larger responses and thus better response SNRs.

### 4.4 Simulated data

The current ABR data set exhibited a wide range of SNRs, from, on average, −8.6 dB for the click-evoked condition high-pass-masked at 0.5 kHz, to 9.8 dB for the broadband chirp-evoked condition. To investigate the effect of SNR more directly, we created a realistic simulated data set with a similar range of SNRs as the measured data. This enabled us to create individual responses with known actual warping functions, and thus directly evaluate the accuracy with which they were estimated.

**Fig. 7:**
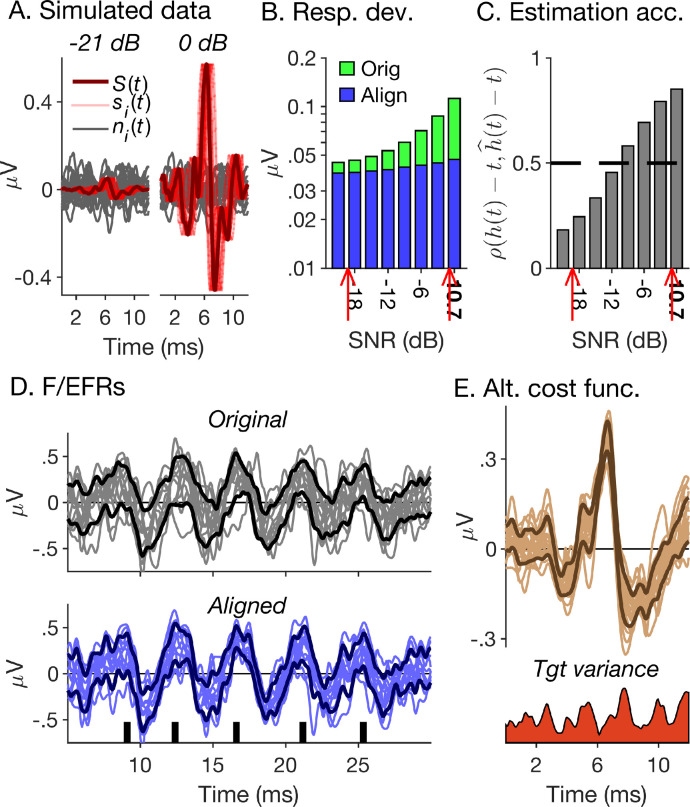
Simulated data and outlook. (A) Simulated ABRs. The dark red line shows the structural response, *S*(*t*), and the lighter red lines, the individual true ABRs, *s*(*t*). The grey lines show the individual noise traces. The left and right panels show the responses for the lowest and highest simulated SNRs (Δ*snr* = −21 and 0 dB), respectively. (B) Response deviations of the original (green bars) and aligned (blue bars) simulated responses as a function of their SNR (expressed as Δ*snr* for all conditions other than Δ*snr* = 0). The red arrows show the range of SNRs of the measured responses. (C) Estimation accuracy of time warping functions, measured as $\rho (h\left(t\right)-t,\hat{h}\left(t\right)-t)$, as a function of SNR. (D&E) Possible future applications and extensions. (D) Non-linear curve registration could also be applied to frequency- or envelope-following responses (F/EFRs). This example shows responses to a short train of chirps, aligned using the *at, PSDD, λ* = 0 registration condition. The original and aligned responses are shown in the top and bottom panels, respectively. As in Fig. 5, the thinner, lighter-coloured lines show the individual responses and the thicker darker-coloured lines show their pointwise standard deviations. (E) In future, the criterion used for fitting the warping functions could take into account the variance as a function of time (bottom panel) of the target response (top panel; here, the aligned responses to the broadband chirp condition, replotted from Fig. 5A).

To create the simulated data, we first stacked the click-evoked ABRs to create larger broadband responses, compensated for cochlear dispersion [63], and then aligned the stacked responses using the *PSDD, at, λ* = 0 registration condition that created the best match between manual and automatic picking results. Then, we averaged the aligned responses across subjects to create a prototypical, or structural response, *S*(*t*) (Fig. 7A), which we de-aligned using the inverse of the individual warping functions, $h_{i}^{-1}\left(t\right)$, to create individual true ABRs, $s_{i}\left(t\right)=S\left(h_{i}^{-1}\left(t\right)\right)$. Finally, we created individual noise traces by taking the plus-minus reference of the replicate responses, $n_{i}\left(t\right)=\left(x_{i}^{\left(1\right)}\left(t\right)-x_{i}^{\left(2\right)}\left(t\right)\right)/2$[62], and then added the true ABRs to the noise traces after multiplying them with factors 10^Δ^*^snr^*^/20^, where Δ*snr* ranged from −21 to 0 dB in 3-dB steps: ${\hat{x}}_{i}\left(t\right)=10^{\Delta snr/20}\cdot s_{i}\left(t\right)+n_{i}\left(t\right)$. The SNRs of the resulting simulated responses, ${\hat{x}}_{i}\left(t\right)$, ranged from −10.3 dB at Δ*snr* = −21 dB to 10.7 dB at Δ*snr* = 0 dB. Like the measured responses, the simulated responses were warped to the condition with the highest SNR (Δ*snr* = 0 dB) using the *PSDD, at, λ* = 0 registration condition. This yielded “estimated” warping functions, ${\hat{h}}_{i}\left(t\right)$, which were compared with the actual warping functions, *h_i_*(*t*), by correlating ${\hat{h}}_{i}\left(t\right)-t$with *h_i_*(*t*) − *t* (Fig. 7C).

Fig. 7B shows that the reduction in response deviation between the original and aligned simulated responses decreased with decreasing SNR. This is expected to the extent that the warping aligned the true ABRs rather than the noise traces. At the same time, the correspondence between the estimated and actual warping functions also decreased with decreasing SNR, from an average of 0.855 at SNR = 10.7 dB to an average of only 0.161 at SNR = −10.3 dB (Fig. 7C). This shows that alignment quality depends strongly on the quality of the to-be-aligned responses: the lower the response SNRs, the more the alignment will be affected by noise. This, however, will also be the case for manual picking, as human observers will pick waveform peaks and troughs, the location and size of which is influenced by noise. This may be why manual and automatic picking results showed good correspondence even for conditions that yielded poor SNRs.

### 4.5 Results summary

In comparing different registration conditions, we found that (i) average-target (*at*) registration generally yielded better results than pairwise (*pw*) registration, (ii) squared-difference minimization generally yielded better results than correlation maximization, and (iii) registering the response derivatives generally yielded better results than registering the responses themselves.

Overall, the *at* registration procedure, combined with squared-difference minimization of the response derivatives (*PSDD*) with no roughness penalty (*λ* = 0) yielded the best match between automatically and manually picked wave-I and -V latencies and amplitudes.

## 5 Discussion

The aim of this study was to develop a procedure for extracting individual latencies and amplitudes of ABR waves with minimal manual involvement. The procedure involves non-linearly aligning, or “warping”, individual ABRs using the methodology of curve registration. To construct smooth and invertible time warping functions, we adopted the computationally efficient “continuous monotone registration” approach proposed by Ramsay [50]. Once aligned, the individual ABRs are averaged to create a structural average response. This is used for picking the required waves’ structural peak and trough latencies, which, in turn, are used to derive their individual latencies and amplitudes.

In the current study, the procedure was applied to align a set of individual ABRs (from multiple subjects and conditions) with one another, and thus the structural average response was constructed from that set. Alternatively, individual ABRs could be aligned with a previously constructed structural average response, based on an independent (e.g., normative) data set, and the respective individual wave latencies and amplitudes could be derived from previously picked (normative) structural peak and trough latencies.

To minimize the danger of over-alignment, any differences in amplitude shift or scale between the to-be-aligned responses should be minimized by appropriate linear transformations. In the current study, individual ABRs were mean-centred and normalized by their root-mean-square amplitude. Similarly, any overall latency differences between the to-be-aligned responses should be accounted for by linear time shifts. In the current study, ABRs were shifted by the latency differences between the subject-average responses for different experimental conditions. In other situations, linear time shifts may be required to account for overall latency differences between different subjects or subject groups.

In the current study, the proposed procedure was applied to an example set of transient-evoked ABRs. There is no reason, however, why it could not also be applied to other types of ABRs, particularly those evoked by longer and more complex sounds, such as speech syllables or short trains of clicks or chirps [64,65]. Such responses typically consist of multiple peaks, which roughly follow the stimulus waveform and are thus commonly referred to as “frequency-” or “envelope-following responses” (FFRs or EFRs; see Fig. 7D, upper panel, for an example). F/EFRs are often evaluated using summary measures, such as the stimulus-to-response correlation or the response frequency spectrum [66], calculated over the entire response duration. Such measures, however, cannot reveal differences between individual response peaks, which may arise as a result of differences between eliciting stimulus portions, or in consequence of neural factors, such as adaptation or efferent feedback [67]. Nonlinear curve registration could be used to evaluate such inter-peak differences (see Fig. 7D, lower panel).

In the current study, all tested criteria for fitting the time warping functions were based on the deviation between the warped and target responses (or response derivatives), without any consideration of their variability. In contrast, warping paths for aligning speech tokens with templates in automatic speech recognition are fitted with consideration of the templates’ segment-wise variability [68]. A similar strategy could also be applied to ABRs, particularly when aligning individual responses with a normative target response. Specifically, the deviation between the warped and target responses could be weighted in inverse proportion to the target variance (see Fig. 7E).

## 6 Appendix: mode of availability of software

The current procedure involves (i) pre-processing of individual ABRs, (ii) alignment of the responses using continuous monotone time warping, and, finally (iii), picking of structural peak and trough latencies and derivation of individual wave latencies and amplitudes. All of these steps have been implemented as separate Matlab functions, preproc.m, nlcurvereg.m and xtractlatamp.m, respectively (written in Matlab version R2019a), which are available for download from https://github.com/mszkk3/Non-linear-ABR-registration-.git. All three functions provide the option of analysing data either from a single experimental condition or from multiple conditions. In addition, they also give different options for defining the warping target. When there is only a single experimental condition, the target is the average response across all individual ABRs. When there are multiple conditions, the user has the option of warping to the subject-average response for a specific condition (like the broadband condition in the current study), or the grand-average response across all subjects and conditions. Alternatively, the responses can also be aligned with a separate target response, which could be based on an independent data set. The pre-processing function, preproc.m, offers the choice of pre-aligning either the subject-average responses across conditions (in the case of multiple conditions), or the individual responses across individuals (in the case of a single condition). The warping function, nlcurvereg.m uses the non-linear problem solver, *fmincon*, which requires Matlab's Optimization toolbox.

In addition, we also provide the current chirp-evoked data in a .mat file (chirpdata.mat) as well as a matlab script with three examples of how the functions’ different options could be used.

## Declaration of Competing Interest

None of the authors declares any conflict of interest.
